# Supplementary material for: Single-molecule characterization of subtype-specific β1 integrin mechanics
Source: Nat Commun. 2022 Dec 3;13:7471. doi: 10.1038/s41467-022-35173-w (PMC9719539; doi:10.1038/s41467-022-35173-w)
Supplement: Supplementary file 1 — Supplementary Information [file 41467_2022_35173_MOESM1_ESM.pdf]

Supplementary Information

Single-Molecule Characterization of Subtype-Specific β1 Integrin Mechanics

Jo & Li et al.

Contents

Supplementary Fig. 1-14

Supplementary Table 1-2

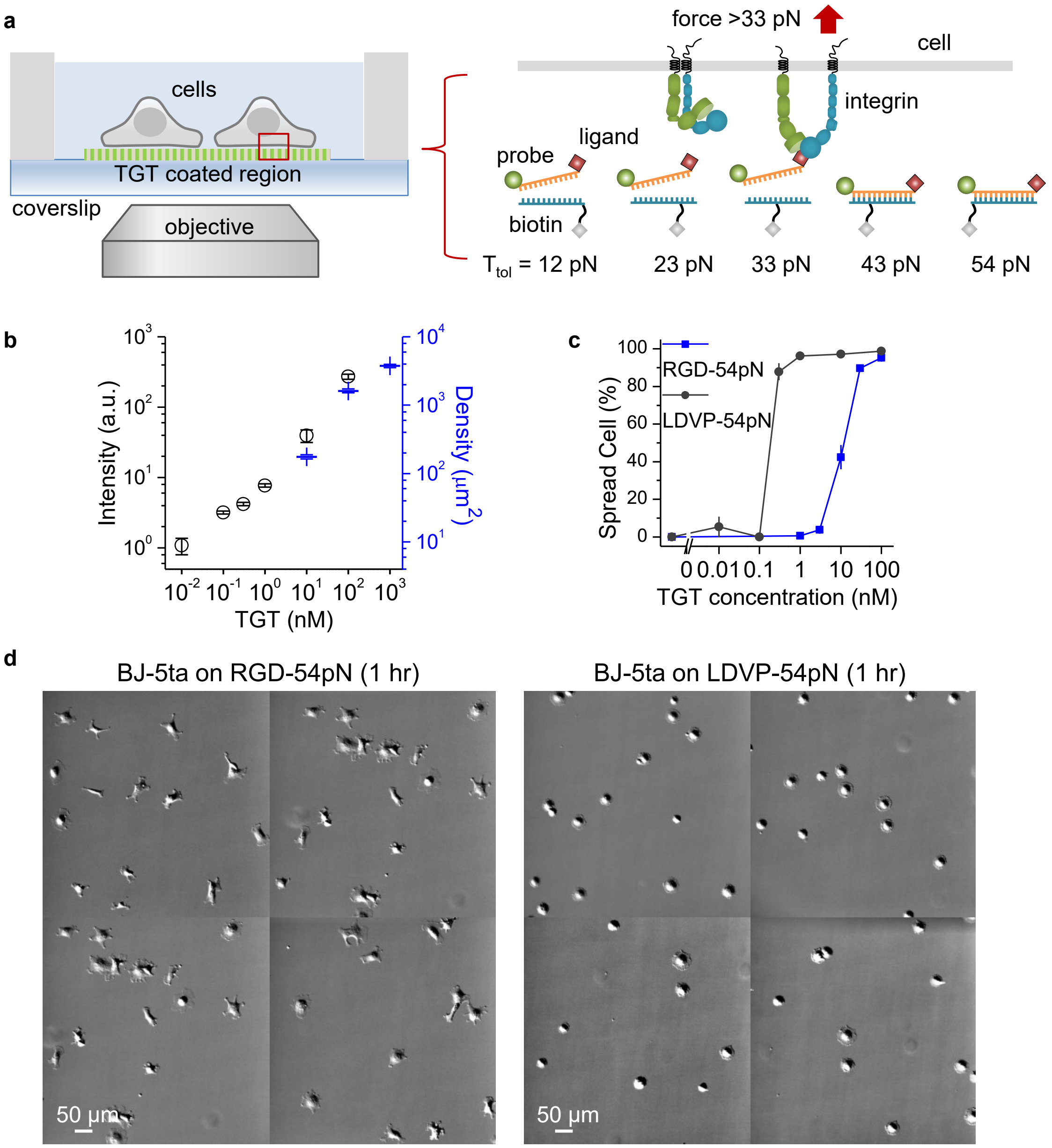

Supplementary Fig. 1. Cells spreading on LDVP or RGD-TGT surface

(a) Schematic of TGT with five different tension tolerances. (b) Fluorescent signal and immobilized TGT density according to TGT concentration on neutravidin coated PEG surfaces (10 min incubation). Fluorescence signals (Cy3) were measured using epifluorescence microscopy (black circles). The linearly increasing intensity shows that biotin-binding sites on the neutravidin coated surface are not saturated up to 100 nM. The data represent the mean ± SD from nine measurements (87 by 87 µm each). The molecular density was measured based on single-molecule detection (blue bars). See Supplementary Fig. 4a. TGTs with and without Cy3 were mixed for 100 nM (10% Cy3-TGT) and 1 µM (1% Cy3-TGT) conditions to use the identical imaging condition with 10 nM (100% Cy3-TGT) condition. The data represent the mean ± SD from five regions (87 by 87 µm each). Arbitrary units (a.u.). (c) Percentage of spread cells on RGD-54pN or LDVP-54pN according to TGT concentration (mean ± SE; n=3 independent experiments). TGT 100 nM was used across this study when the concentration is not specified. (d) Representative differential interference contrast (DIC) images (10X) of cells seeded on RGD-54pN (left) or LDVP-54pN (right) for 1 hr. Scale bar, 50 µm. Source data are provided as a Source Data file.

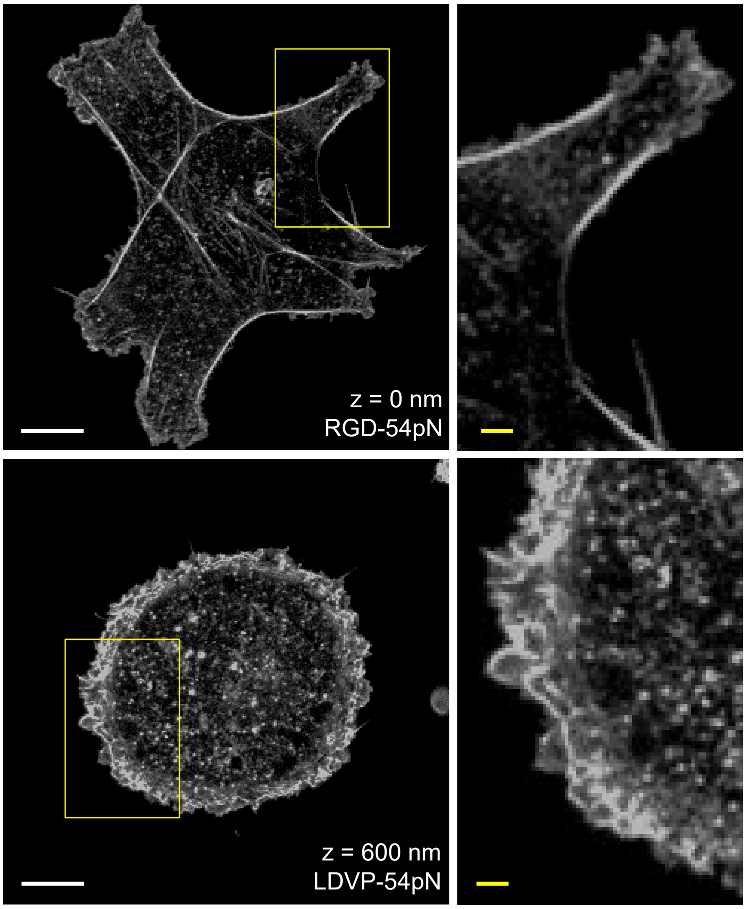

Supplementary Fig. 2. Confocal images of cells spreading on LDVP or RGD-TGT

Confocal images of phalloidin (AF555) show cytoskeletal structures of spreading cells. BJ-5ta fibroblasts were fixed after 1 hr spreading on RGD-54pN (top) and LDVP-54pN (bottom). Boxed regions are magnified in the right column. Scale bars: 10 μm (white), 2 μm (yellow).

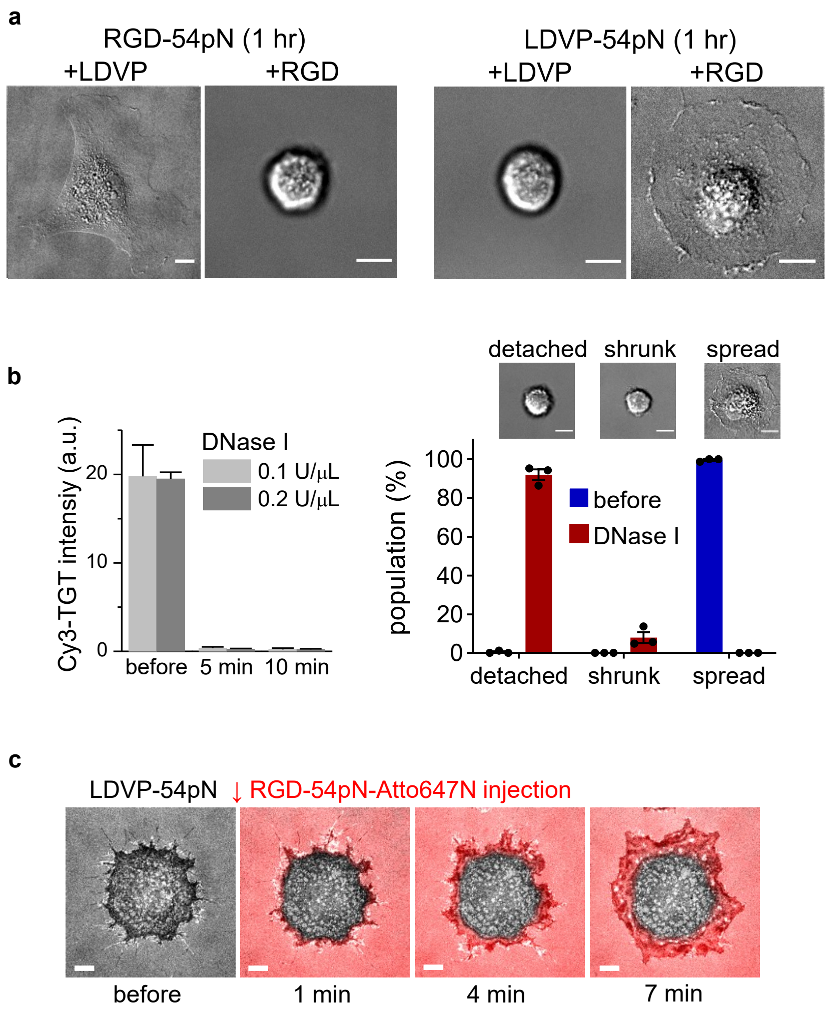

Supplementary Fig. 3. RGD or LDVP-TGT mediated cell spreading

(a) DIC images (60X) of cells seeded on TGT surfaces in the presence of soluble LDVP (100 μM) or RGD (100 μM) for 1 hr. Scale bars, 10 μm. (b) To show that cell spreading is supported by TGT, DNase I was injected to the chamber with cells spreading on LDVP-54pN. Cy3 intensity of TGT surface shows the relative TGT density before and after DNase I treatment (left panel, mean ± SD, n=25 loci). The percentage of cell shape before and 5 min after DNase I treatment (right panel, mean ± SE, n=3 independent experiments). Cells were efficiently detached from the surface or shrunk with small contact. Arbitrary units (a.u.). (c) Additional immobilization of RGD-54pN. Cells were seeded on LDVP-54pN-Cy3 for 1 hr and RGD-54pN-Atto647N was injected (100 nM). The merged image is the overlay of RICM and Atto647N signal (red, TIRFM) on the surface. Scale bar, 10 μm. Source data are provided as a Source Data file.

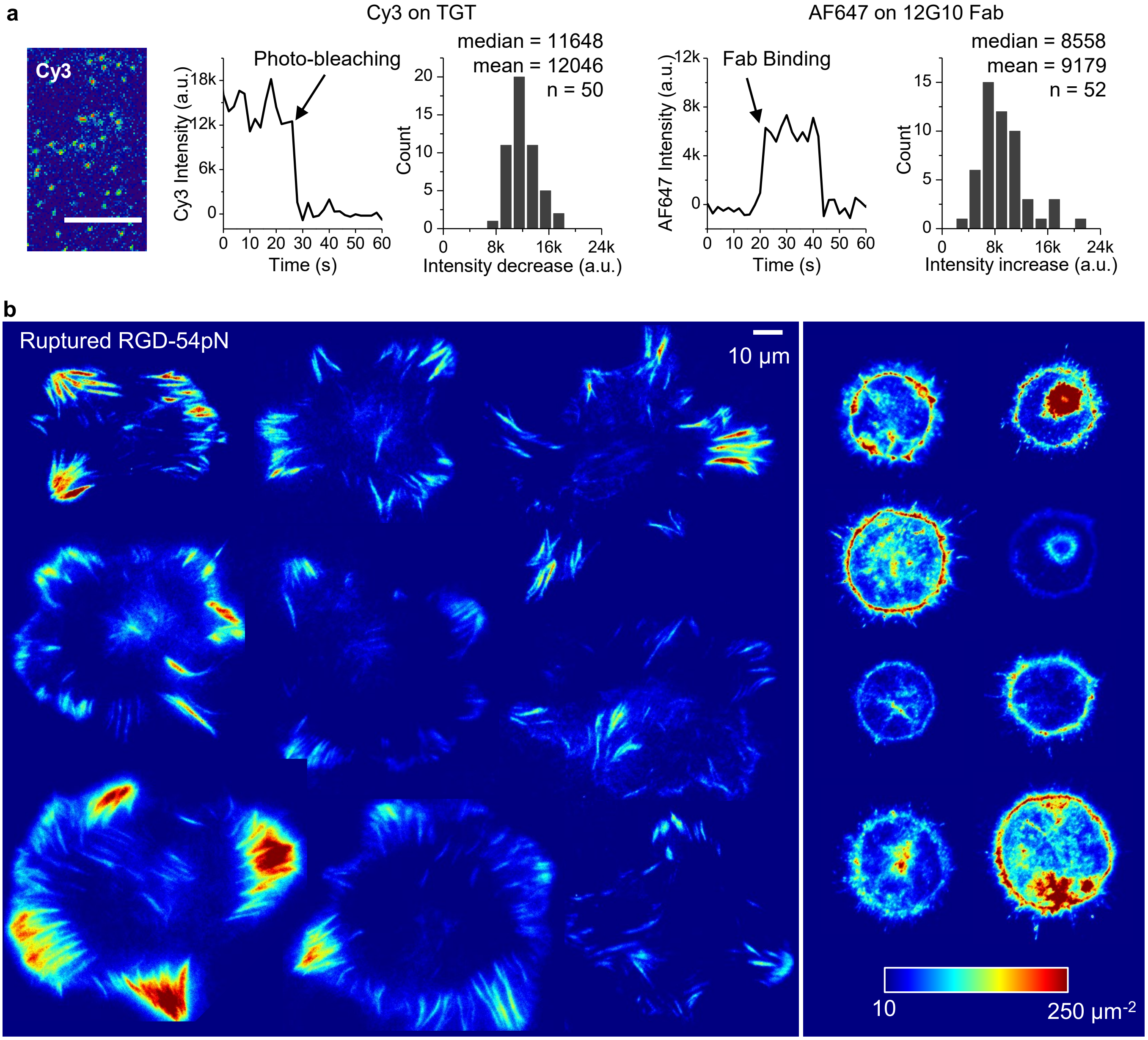

Supplementary Fig. 4. High force transmission events recorded by qTGT.
(a) Calibration of fluorescence signal for TGT, ruptured qTGT and bound Fab density. Fluorescent intensity was converted to the probe density by analyzing stepwise change of single-molecular signals. TIRF imaging was done for the region whose TGT or qTGT density is low enough to distinguish single-molecules (left image) on the same coverslip. The identical imaging condition was used except the exposure time (2 s for single-molecule imaging, 0.1-0.5 s for data acquisition). Scale bar, 10 μm. A time trace of Cy3 signal intensity with a photobleaching event is shown. The representative distribution of Cy3 signal intensity from a calibration is shown. The median was used for TGT density calibration. For fluorescently labeled Fab (12G10 Fab, AF647), non-specific transient Fab binding events to a surface were monitored and analyzed in the similar way. The mean of the stepwise signal increase was used for the calibration considering multiple dye conjugated molecules (mostly 1-2 dyes). 40 or more events were collected for each calibration. This calibration was done for each experiment. Arbitrary units (a.u.). (b) Calibrated ruptured qTGT density maps (see color key, μm^{-2}) for a gallery of representative cells seeded on RGD-54pN (left) or LDVP-54pN (right). Scale bar, 10 μm.

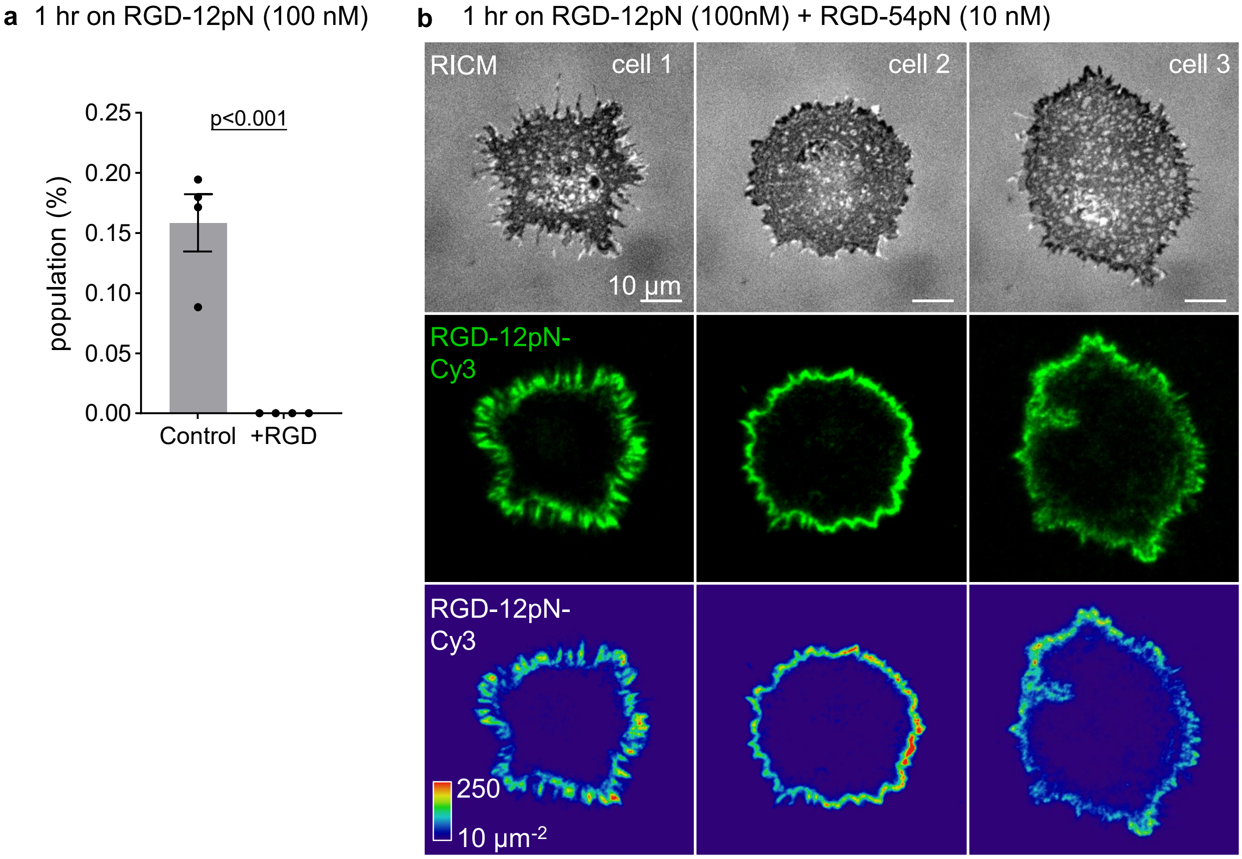

Supplementary Fig. 5. Low force transmission events recorded by qTGT.

(a) The percentages of cells that produced rupture signals. BJ-5ta cells were seeded on RGD-12pN (BHQ2-Cy3, 100 nM) in the absence or presence of soluble cRGDfK ligand (100 μM; mean ± SE, n = 4 independent experiments; 36, 34, 35, 50, 44, 26, 25, 16 cells for each set). No cell produced a significant rupture signal in the presence of soluble cRGDfK. Two-sided t-test for p-values.

(b) BJ-5ta cells were seeded on the surface with two different TGTs (RGD-12pN-BHQ2-Cy3 100 nM and RGD-54pN-BHQ2-Atto647N 10 nM). Ruptured RGD-12pN signals are shown in green (middle row) and the calibrated density is shown in the bottom row (see color key). Because cells do not spread on RGD-12pN surfaces, a small amount of RGD-54pN was added to facilitate cell spreading. In this mixed condition, cells adhered to the surface and spread, but the spreading is limited compared to that of cells on high density RGD-54pN. Abundant Cy3 signals from ruptured RGD-12pN were observed near the leading edge and filopodia. The calibrated molecular density maps show that the ligand of RGD-12pN was pulled by cells frequently. See color key for molecular density. The rupture signal of the RGD-54pN TGT (Atto647N) was negligible in this condition. Source data are provided as a Source Data file.

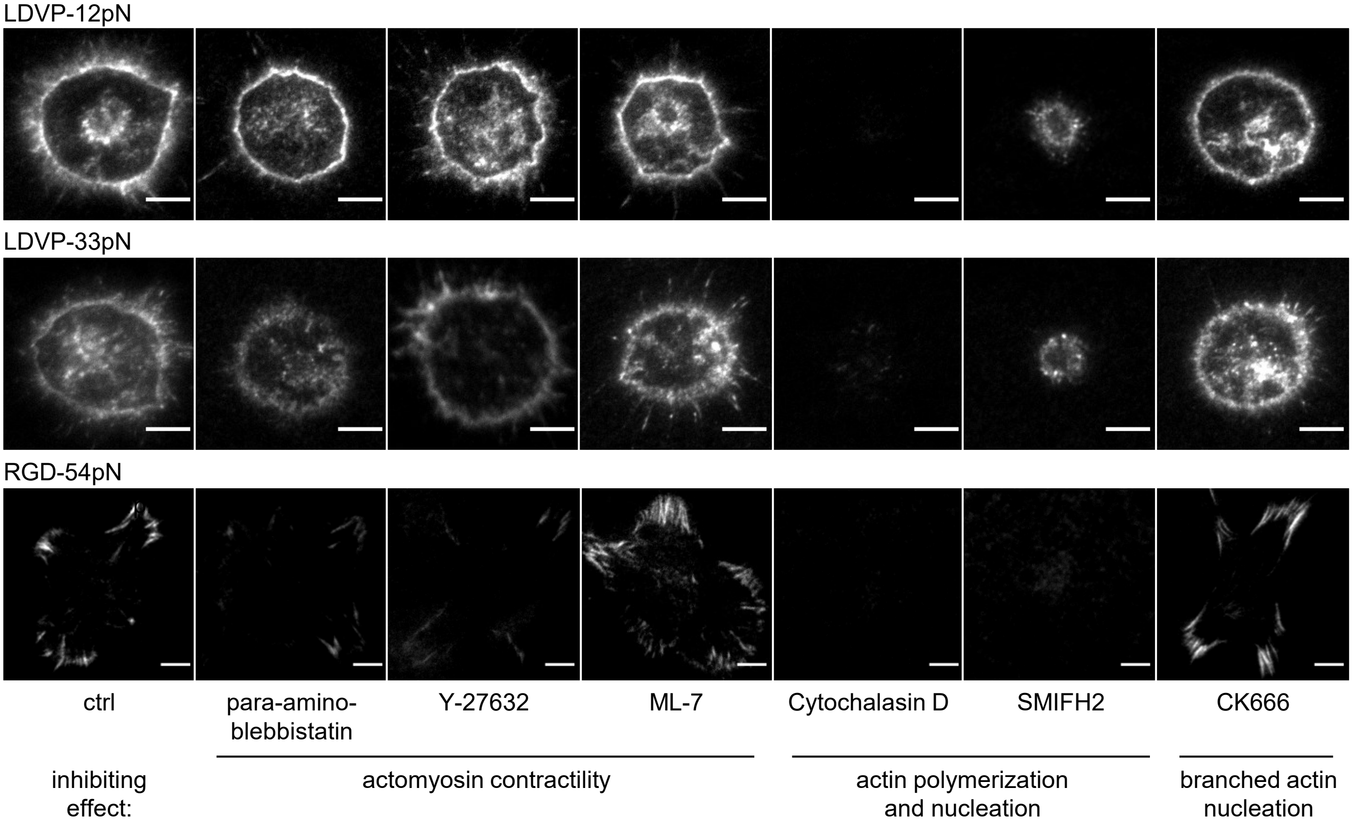

Supplementary Fig. 6. Cytoskeletal inhibitor effects on TGT rupture

Representative images of LDVP-12pN, LDVP-33pN and RGD-54pN (BHQ2-Cy3) ruptured by cells seeded for 1 hr in presence of cytoskeletal inhibitor (5 min preincubation): para-amino-blebbistatin (50 uM), Y-27632 (10 μM), ML-7 (10 μM), Cytochalasin D (10 μM), SMIFH2 (50 μM), and CK666 (50 μM). The outermost rupture signals reflect the spread cell area. Cells did spread when Cytochalasin D or SMIFH2 was added. Scale bar, 10 μm

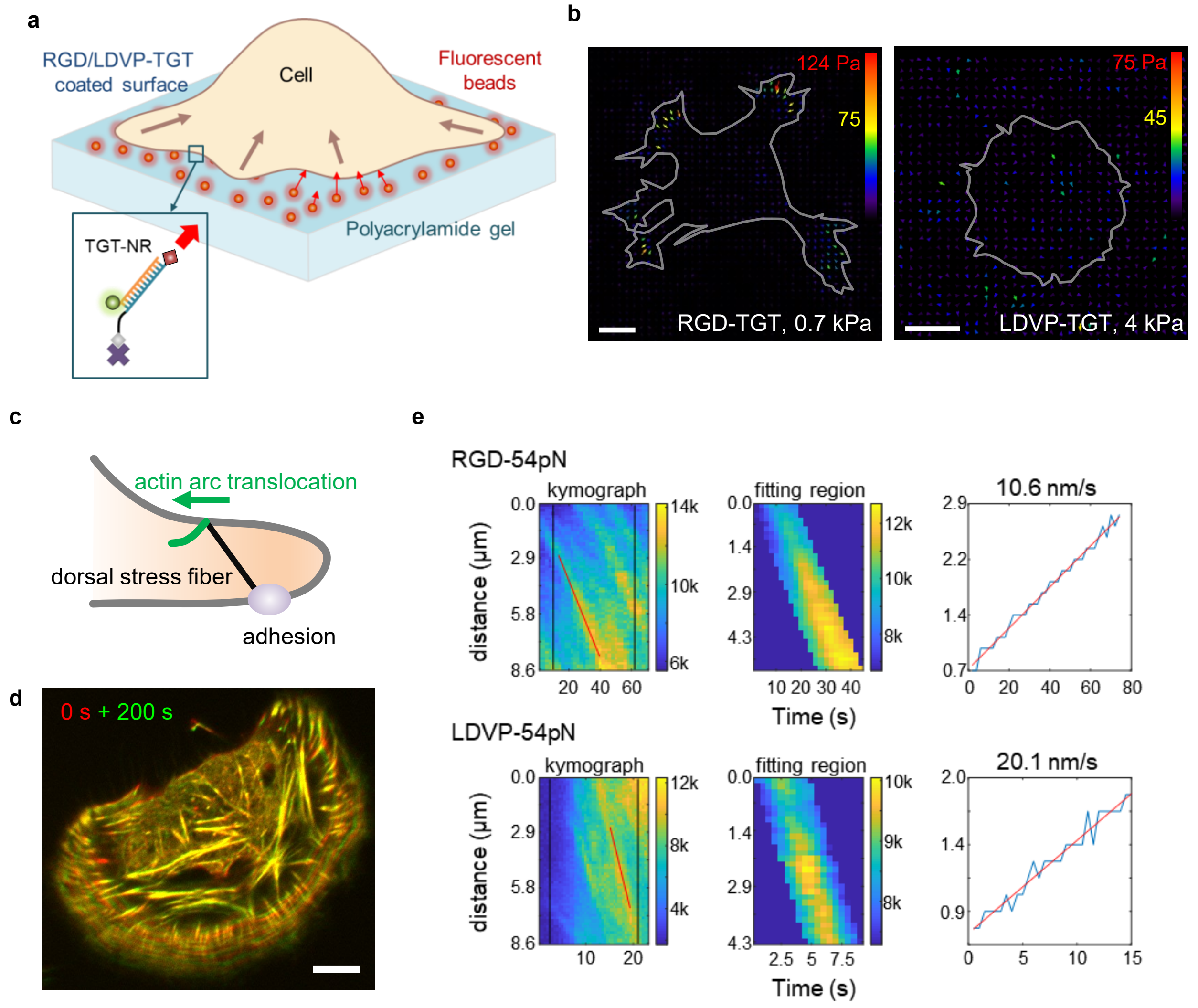

Supplementary Fig. 7. Traction force and retrograde flow rate measurement.
(a-b) Traction force of BJ-5ta fibroblasts on RGD or LDVP. (a) Schematic of traction microscopy using non-rupturable, TGT-like DNA linkers (Supplementary table 2). Ligands were conjugated on the biotinylated strands to prevent detachment of ligands from the surface. (b) Representative mechanical stress maps (see color key). Gray lines indicate the cell outlines. Scale bar, 10 μm. (c-e) Flow speed of actin arc was measured analyzing time-lapse TIRFM images of Sir-actin (10 or 20 nM). (c) Schematic of actin arc translocation connected to the adhesion. (d) Two time points were overlaid for a cell on RGD-54pN with pseudo-color to show the actin movement over time (red for 0 s and green for 200 s). Scale bar, 10 μm. (e) Representative kymographs for actin arc translocation analysis. The red lines indicate the spatiotemporal regions for linear fitting

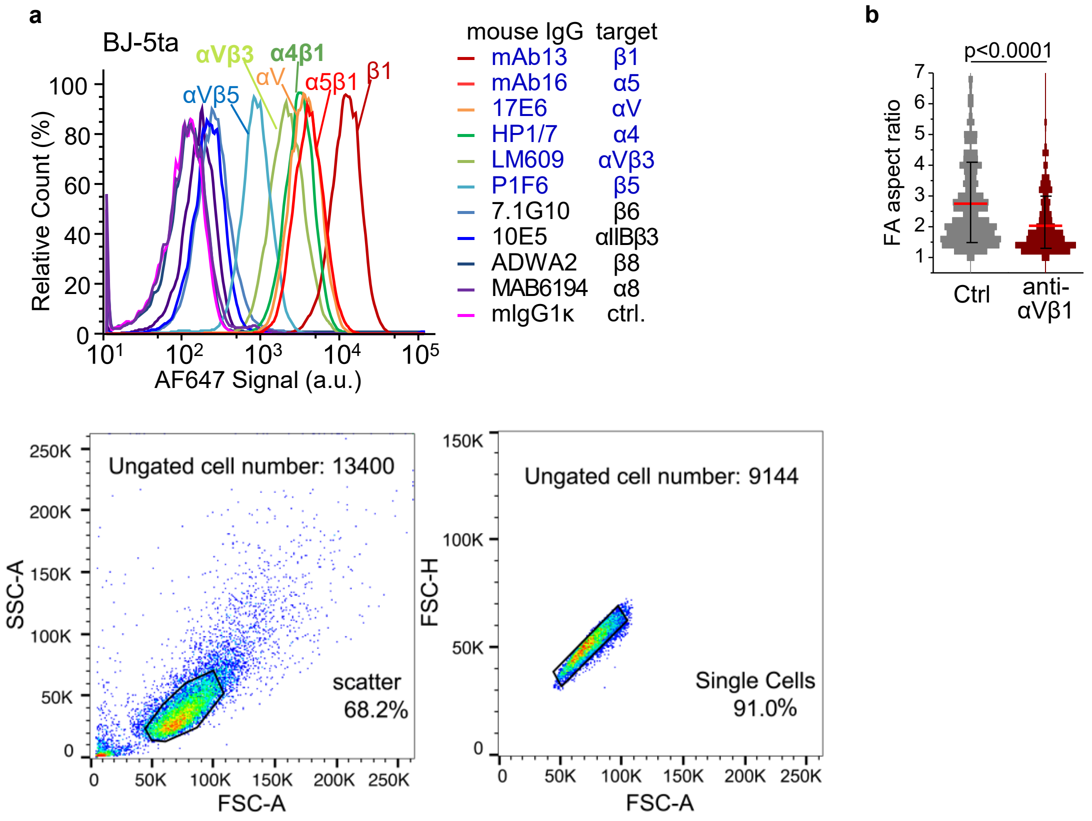

Supplementary Fig. 8. Expression of integrins on BJ-5ta fibroblasts

(a) Immuno-fluorescent flow cytometry of RGD-binding integrins and integrin α4β1 on BJ-ta cells using indicated mouse IgGs and AF647-labeled goat anti-mouse IgG. Antibodies with positive labeling are named in blue on right. Single cells were selected for analysis based on FSC-A/SSC-A scattering and FSC-A/FSC-H gating in all FACS data in this paper, and an example is shown (lower panels). (b) Aspect ratio of focal adhesions of cells seeded on RGD-54pN for 1 hr in absence or presence of inhibitory Biogen-αVβ1.5 Fab to αVβ1. Paxillin was immunostained (AF488) as the focal adhesion marker (clusters >1.2 μm^2; mean ± SD; n = 2116 and 2251 from 13 and 17 cells). Two-sided t-test for p-values. Source data are provided as a Source Data file.

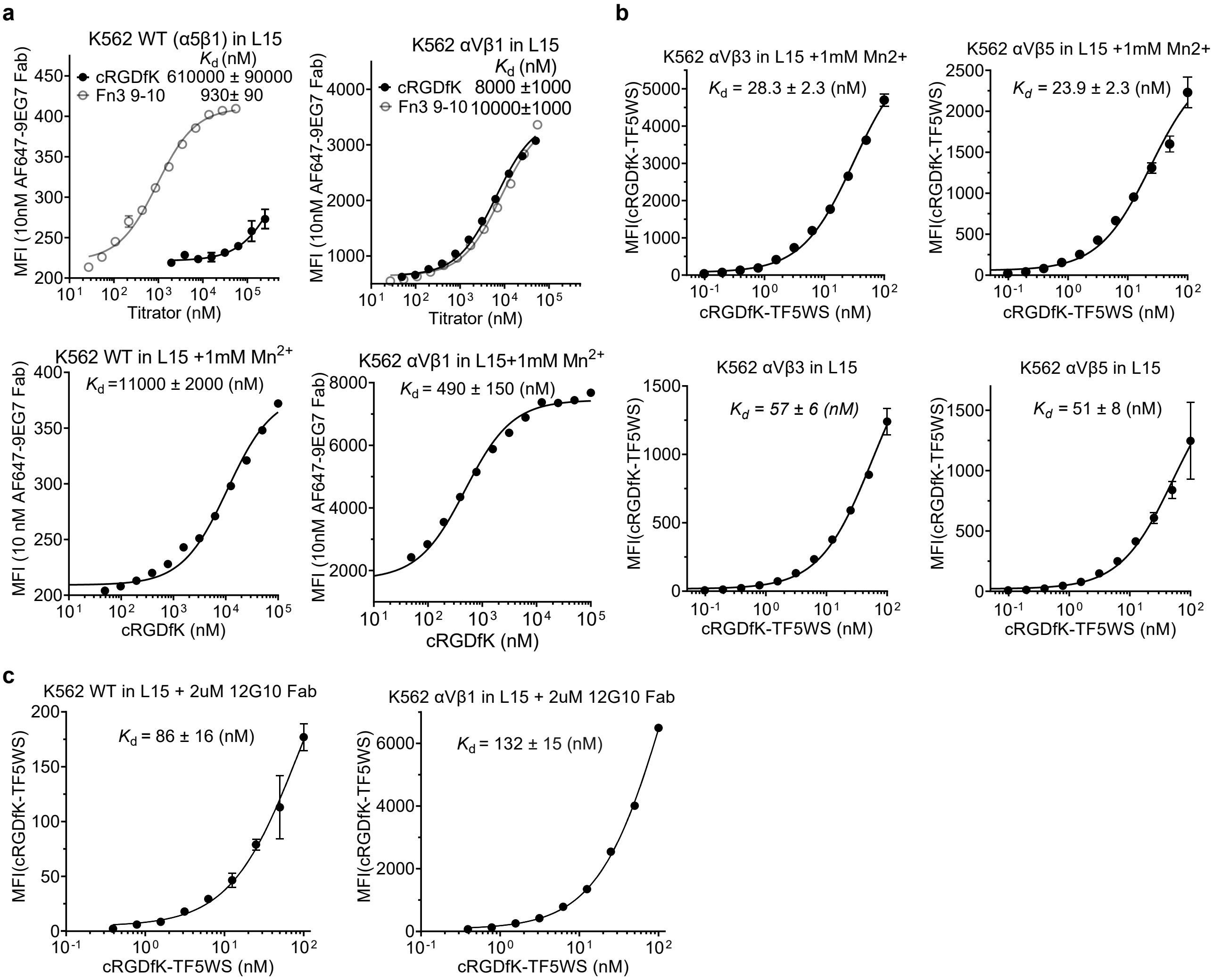

Supplementary Fig. 9. Binding affinity of cRGDfK for RGD-binding integrins on cell surfaces.

Affinities of cRGDfK peptide to intact integrins on K562 cells (wildtype which expresses α5β1; αVβ1, αVβ3, and αVβ5 stable transfectants) in L15 medium containing 1% BSA were measured by flow cytometry without washing. (a) Affinities of cRGDfK to α5β1 on native K562 cells and αVβ1 on K562 transfectants were measured by enhancement of binding of AF647-9EG7 Fab (10 nM), which is specific for the β1 extended conformation. Mean fluorescence intensity (MFI) at each cRGDfK peptide concentration was fitted to a dose-response curve with three parameters (background MFI, maximum MFI, and the EC50 value which represents the K_d value of cRGDfK peptide to the respective integrin). In L15 without Mn^{2+}, macromolecule fragment Fn3 9-10 was included as a titrator to better determine the maximum MFI. The background and maximum MFI values were shared for cRGDfK and Fn3 9-10 fitting. Binding to the natively expressed α5β1 in αVβ1 transfectants was negligible compared to αVβ1, owing to the much higher expression of αVβ1, as shown by the much lower affinity for fibronectin and much higher affinity for cRGDfK in the αVβ1 transfectants compared to wildtype K562 cells in absence of Mn^{2+} (upper panels), as well as the much higher affinity for cRGDfK in the αVβ1 transfectants compared to wildtype K562 cells in presence of Mn^{2+} (lower panels). (b) Affinity of cRGDfK to intact αVβ3 and αVβ5 was measured on K562 αVβ3 and K562 αVβ5 stable transfectants, respectively, by saturation binding of cRGDfK peptide with lysine side chain conjugated with TideFluor5WS (TF5WS) (custom synthesized by BACHEM). Background MFI was measured in the binding buffers with 10 mM EDTA. The background-subtracted MFI at each cRGDfK-TF5WS concentration was fitted to a dose-response curve with three parameters (background MFI, maximum MFI at saturation binding level, and K_d value of cRGDfK-TF5WS to integrin αVβ3 or αVβ5). Binding to the natively expressed α5β1 in αVβ3 and αVβ5 transfectants was negligible, owing to the much higher expression of αVβ3 and αVβ5, respectively on the transfectants, and non-detectable specific MFI of cRGDfK-TF5WS (100nM) on wildtype K562. (c) Intrinsic affinity of cRGDfK-TF5WS for EO state of integrin α5β1 or αVβ1 stabilized by 2 μM 12G10 Fab was measured using same methods as in (b). The error bars indicate the difference of MFI from two independent measurements. Errors of K_d are standard error (SE) from least-square fitting, and the 95% confidence intervals are obtained as fitting values ± 1.96×SE. Source data are provided as a Source Data file.

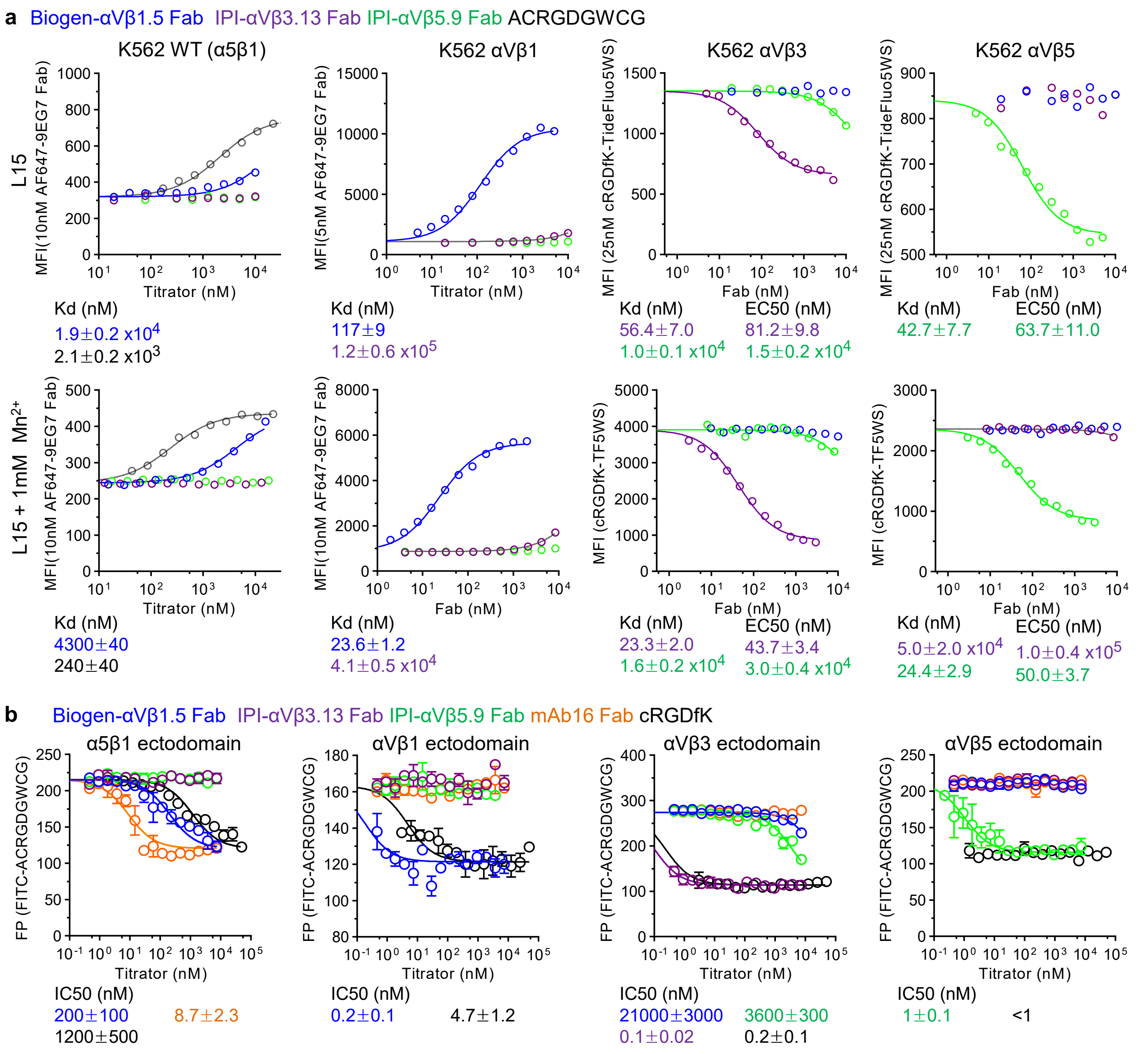

Supplementary Fig. 10. Affinity and specificity of integrin inhibiting Fabs.

(a) Affinities of RGD-mimetic Fabs to intact integrins on K562 cells in L15 medium containing 1% BSA with or without 1mM Mn^{2+}, quantified by flow cytometry without washing. Fab affinities for α5β1 expressed on K562 wild type cells and for αVβ1 expressed on K562 αVβ1 stable transfectants were quantified by enhancement of 10 nM AF647-9EG7 Fab binding. For measurement on α5β1, its high affinity binding peptide, cyclic-ACRGDGWCG, was also included as a titrator to better determine maximum MFI when all α5β1 is in the ligand bound EO state. MFI at each concentration of different titrators was globally fitted to three parameter dose- response curve, with background MFI and maximum MFI as shared fitting parameters, and K_d value for each titrator as individual fitting parameter. Affinity of Fabs to integrin αVβ3 and αVβ5 were determined by competing with 25nM cRGDfK-TF5WS binding on K562 αVβ3 and K562 αVβ5 stable transfectants, respectively. MFI of cRGDfK-TF5WS at each concentration of different competitors were globally fitted to three parameter dose-response curve, with maximum MFI in absence of competitor and minimum background MFI as shared fitting parameters, and EC50 value for each competitor as individual fitting parameter. With the fitted EC50 value, K_d of each competitor was calculated as $K_d = EC50 / (1+ C_L/K_{d,\,L})$, where C_L is the concentration of cRGDfK-TF5WS used (25 nM), and $K_{d,\,L}$ is the binding affinity of cRGDfK-TF5WS to the respective integrin determined in Supplementary Fig. 9 under corresponding condition. (b) Specificity of inhibiting Fabs used in this study checked on soluble integrin ectodomains. IC50s of Fabs were quantified by competing 10 nM FITC-cyclic-ACRGDGWCG peptide binding to 50 nM α5β1, 200 nM αVβ1, 20 nM αVβ3 and 20 nM αVβ5 ectodomains, respectively, by fluorescence polarization (FP) assays. FITC-cyclic-ACRGDGWCG was labeled with FITC at the amino group of the same 6-aminohexanoic acid spacer used for TGT DNA strand attachment. Curves in each panel were globally fitted with three parameter dose response curve, with maximum FP value in absence of titrator and minimum FP value when all the integrin in solution is bound by titrator as shared fitting parameters, and IC50 as individual fitting parameter for each titrator. The error bars indicate the difference of MFI from two independent measurements. Errors of K_d are standard error (SE) from least-square fitting, and the 95% confidence intervals are obtained as fitting values ± 1.96×SE. Source data are provided as a Source Data file.

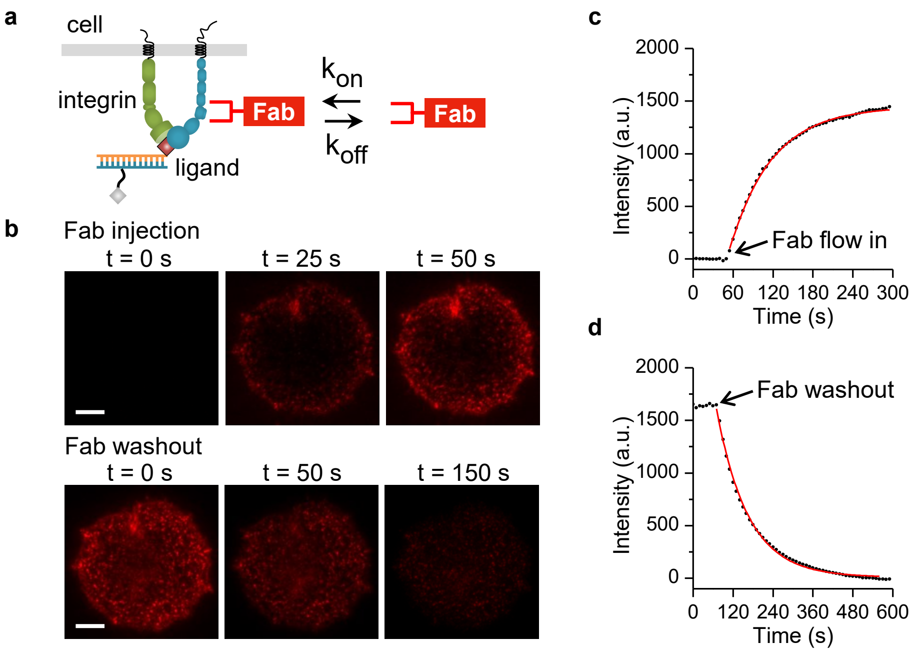

e

Target	Fab	[Fab]	Cell	TGT	k_{obs} (s^{-1})	k_{off} (s^{-1})	k_{on} (μM^{-1} s^{-1})	K_D (nM)
β1E	9EG7-AF647	20 nM	BJ-5ta	LDVP-54pN	0.0146 ± 0.0006	0.0094 ± 0.0003	0.257 ± 0.019	37.3 ± 2.0
β1EO	12G10-AF647	20 nM	BJ-5ta	RGD-54pN	0.0043 ± 0.0004	0.0030 ± 0.0003	0.066 ± 0.010	50.6 ± 8.3
α4	HP1/7-AF488	100 nM	BJ-5ta	LDVP-54pN	0.0130 ± 0.0007	0.0050 ± 0.0002	0.080 ± 0.007	64.8 ± 7.3
αv	13C2-AF488	20 nM	BJ-5ta	RGD-54pN	0.0099 ± 0.0005	0.0049 ± 0.0005	0.250 ± 0.015	20.3 ± 3.1

Supplementary Fig. 11. Fab binding kinetics.

(a) Kinetics were measured with Fabs specific to integrin α-subunits or conformational states of the β1 subunit. To check whether Fab binding kinetics were fast enough for live cell imaging, binding and dissociation rates of Fab were measured on cells spreading on RGD-54pN or LDVP-54pN. Cells were seeded in a flow chamber connected to a syringe pump. Fluorescent Fab solution was injected to measure binding kinetics and subsequently washed out to measure dissociation using TIRFM. (b) Representative images show the binding and dissociation of AF647-labeled 9EG7 Fab. Scale bar, 10 μm. (c-d) Representative association (c) and dissociation (d) curves of 9EG7 Fab on a single cell. The single-exponential fitting values were used for k_{obs} and k_{off}. Arbitrary units (a.u.). (e) Measured and calculated binding kinetic parameters. k_{obs} = (binding time)$^{-1}$. k_{off} = (dissociation time)$^{-1}$. k_{on} = (k_{obs} - k_{off})/[Fab]. [Fab] is the concentration of Fab injected. K_D = k_{on}/k_{off}. Values are mean ± SE of six measurements (two measurements from three cells). Source data are provided as a Source Data file.

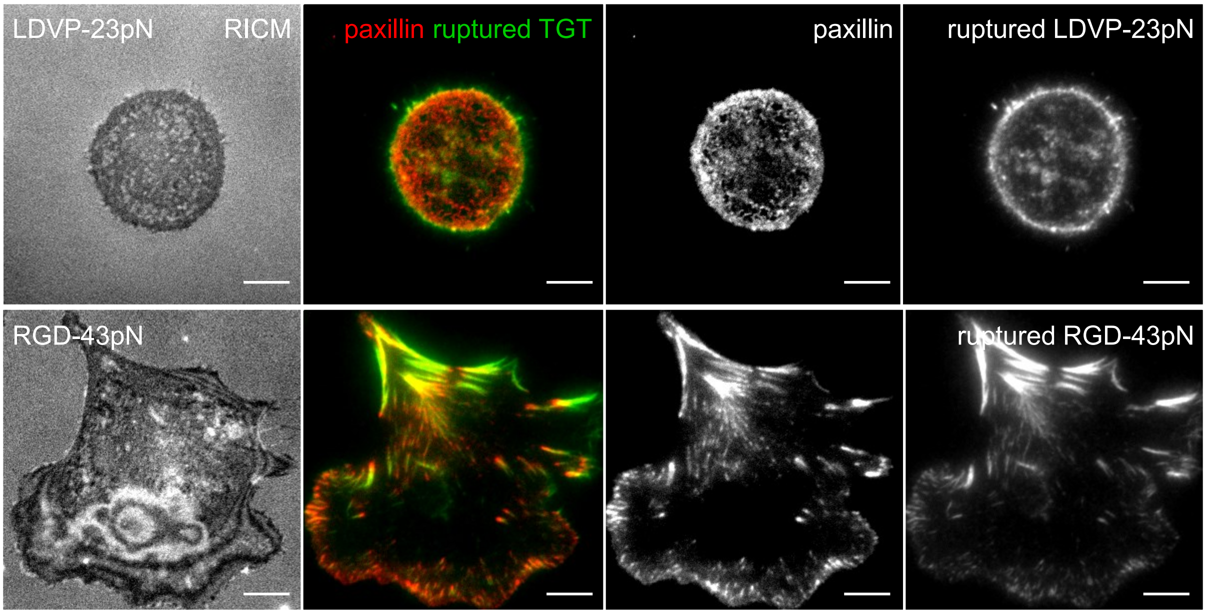

Supplementary Fig. 12. Paxillin recruited near the force transmitting region.
Paxillin-TagGFP2 lentivirus transfected BJ-5ta cells were seeded on LDVP-23pN (upper panels) or RGD-43pN (lower panels) with BHQ2 and Cy3 labeled on the ligand strand and immobilization strands, respectively. TIRFM images of paxillin (red in the 2nd column) and ruptured TGT (green in the 2nd column) are shown along with RICM images after one hour cell spreading. Scale bars, 10 μm.

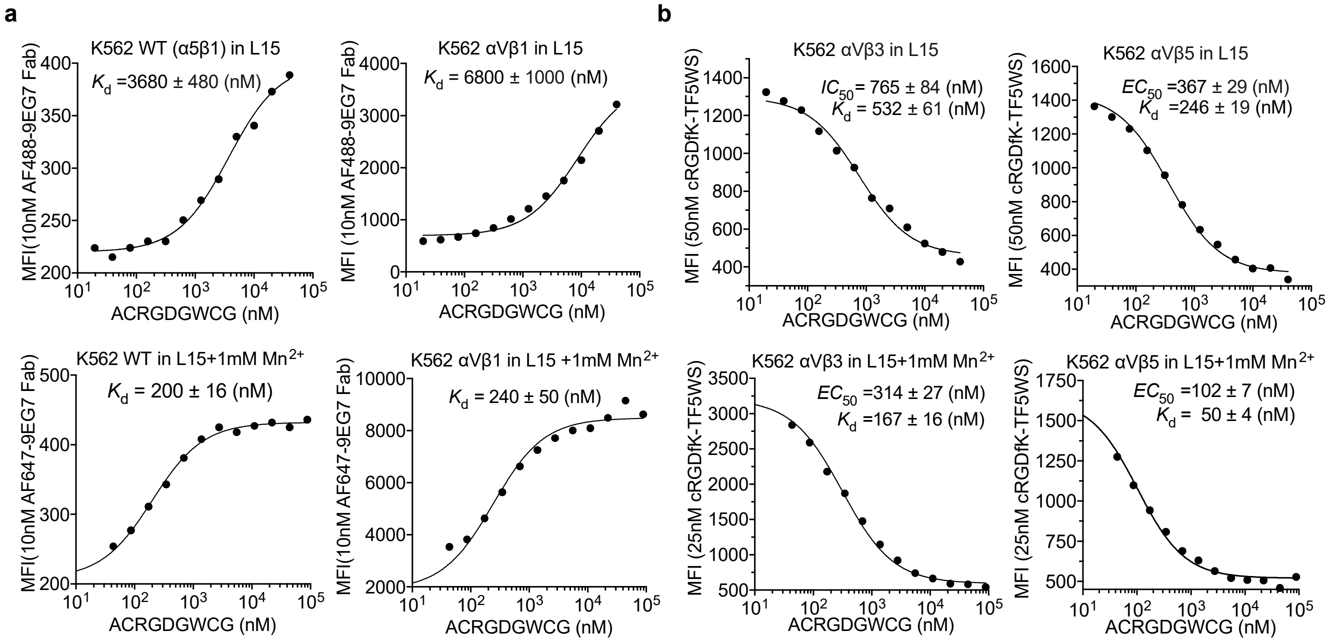

Supplementary Fig. 13. Binding of cyclic-ACRGDGWCGK for RGD-binding integrins on cell surfaces.

Affinities of cyclic-ACRGDGWCGK peptide (ACRGD) to intact integrins on K562 cells (wildtype which expresses α5β1; αVβ1, αVβ3, and αVβ5 stable transfectants) in L15 medium containing 1% BSA were quantified by flow cytometry without washing. (a) Affinities to α5β1 on wildtype K562 cells and αVβ1 on K562 stable transfectants were measured by enhancement of binding of AF647-9EG7 Fab (10 nM), which is specific for the β1 extended conformation. Mean fluorescence intensity (MFI) at each ACRGD concentration was fitted to a dose-response curve with three parameters (background MFI, maximum MFI, and EC50 value which represents the K_d value of ACRGD to the respective integrin). Binding to the natively expressed α5β1 in αVβ1 transfectants was negligible compared to αVβ1, owing to the much higher expression of αVβ1 on the stable transfectants, as also evidenced by the ~10-fold lower binding affinity of Fn3 9-10 to αVβ1 transfectants than native K562 cells (Supplementary Fig. 9a). (b) Affinity of ACRGD to intact αVβ3 and αVβ5, were measured on K562 αVβ3 and K562 αVβ5 stable transfectants, respectively, by competing with cRGDfK-TF5WS peptide binding. MFI of cRGDfK-TF5WS at each concentration of a competitor (ACRGD) was fitted to dose-response curves with three parameters (background MFI, maximum MFI in absence of the competitor, and EC50 value for the competitor). With the fitted EC50 value, K_d of ACRGD was calculated as $K_d = EC50 / (1+ C_L/K_{d,L})$, where C_L is the concentration of cRGDfK-TF5WS used and $K_{d,L}$ is the binding affinity of cRGDfK-TF5WS to the respective integrin determined in Supplementary Fig. 9 under the corresponding condition. The contribution of natively expressed α5β1 to the MFI of cRGDfK-TF5WS on αVβ3 or αVβ5 transfectants was negligible, owing to the much higher expression level of αVβ3 or αVβ5 in the respective transfectants, and the much lower binding affinity of cRGDfK peptide for α5β1 (Supplementary Fig. 9). Thus, competitive binding monitored by the MFI of cRGDfK-TF5WS on αVβ3 and αVβ5 transfectants measures the binding of the competitor (ACRGD) to αVβ3 and αVβ5, respectively. The error bars indicate the difference of MFI from two independent measurements. Errors of K_d are standard error (SE) from least-square fitting, and the 95% confidence intervals are obtained as fitting values $\pm$ 1.96$\times$SE. Source data are provided as a Source Data file.

cRGDfK

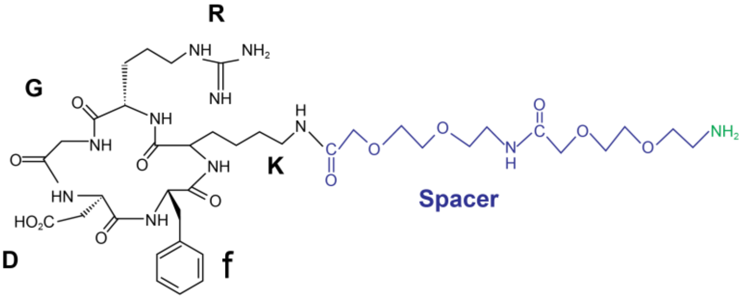

MUPA-LDVPAAK

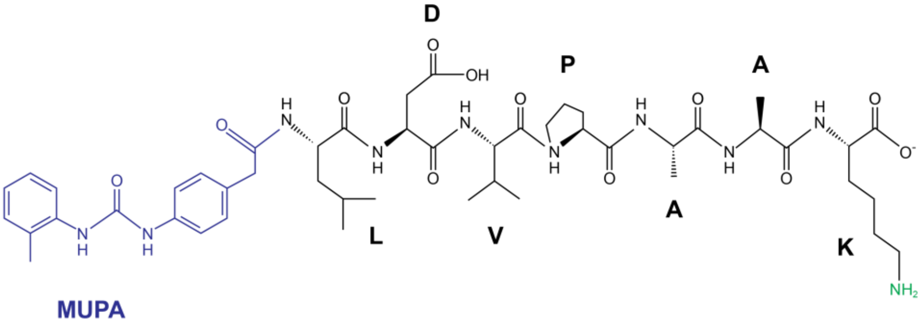

Cyclic-ACRGDGWCGK

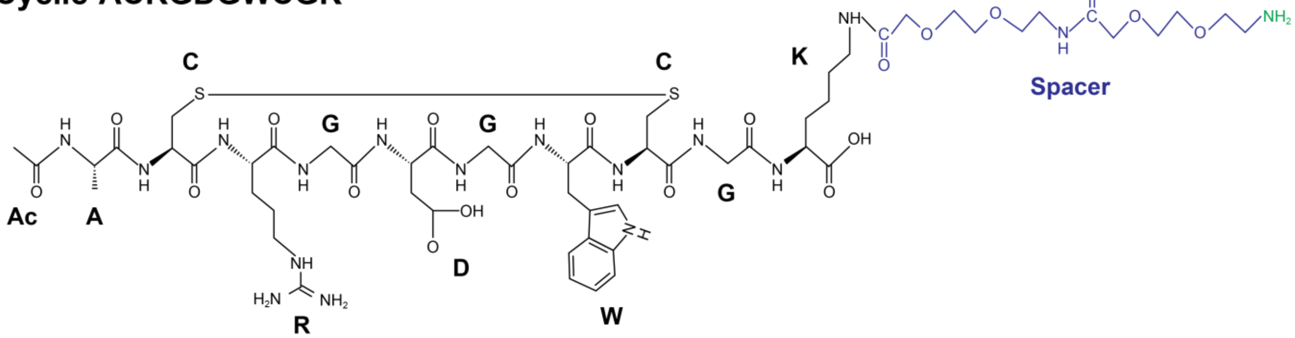

Supplementary Fig. 14. Structural Formulas of peptidomimetic ligands used for TGTs.
Chemical modifications of peptides and DNA attachment sites are colored in blue and green, respectively.

	Heavy-chain CDR3 sequences
IPI-α5β1.2	APGGSVYG
IPI-α5β1.4	QRGLLRPAYG
IPI-αVβ3.7	RVSNSARGDVRVGY
IPI-αVβ3.13	REHIAGRLDDVYYY
IPI-αVβ5.9	AFVRWRGDSLVLSTW
IPI-αVβ5.10	FLGFGRY
Biogen-αVβ1.5	GGPTRGDGTRVYYYGMDV
Biogen-αVβ1.9	GLWSTEVRYYYMDV
Biogen-αVβ1.10	GLWSTEVRYYYMDV

Supplementary Table 1. Heavy-chain CDR3 sequences of the synthetic antibodies.
CDR3 sequences of the synthetic antibodies used in this study. RxD motif is highlighted in gray.

Description	DNA sequence (5’ to 3’)
• Ligand strands for TGT (dye labeled)	
Cy3-labeled	/5Cy3/GGC CCG CAG CGA CCA CCC/3ThioMC3-D/
for Atto647N-labeling	/5AmMC6/GGC CCG CAG CGA CCA CCC/3ThioMC3-D/
• Ligand strand for quenched TGT	
RGD and LDVP-TGT	/BHQ-2/GGC CCG CAG CGA CCA CCC/Thiol C6 SS/
ACRGD-TGT	/BHQ-2/GGC CCG CAG CGA CCA CCC/Amino C7/
• Immobilization strands for TGT (not labeled)	
12 pN	/5AmMC6/GGG TGG TCG CTG CGG GCC
23 pN	GGG /iAmMC6T/GG TCG CTG CGG GCC
33 pN	GGG TGG /iAmMC6T/CG CTG CGG GCC
43 pN	GGG TGG TCG C/iAmMC6T/G CGG GCC
54-pN	GGG TGG TCG CTG CGG GCC/3AmMO/
• Immobilization strands for quenched TGT (dye labeled):	
12 pN	/5Biosg/TTT TTT GGG TGG TCG CTG CGG GCC/3Cy3Sp/
23 pN	GGG /iAmMC6T/GG TCG CTG CGG GCC/3Cy3Sp/
33 pN	GGG TGG /iAmMC6T/CG CTG CGG GCC/3Cy3Sp/
43 pN	GGG TGG TCG C/iAmMC6T/G CGG GCC/3Cy3Sp/
54-pN	GGG TGG TCG CTG CGG GCC /iAmMC6T/TT TTT/3Bio/
• Immobilization strands for non-rupturable TGT-like linker (used for traction force microscopy):	
Non-rupturable	/5AmMC6/GG GTG GTC GCT GCG GGC C/3ThioMC3-D/
/ThioMC3-D/ or /Thiol C6 SS/, disulfide modifier with C3 or C6 linker (for ligand conjugation);<br>/Amino C7/, amino modification with C7 linker (for ligand conjugation);<br>/5AmMC6/ or /3AmMO/ amino modification (for biotinylation);<br>/iAmMC6T/, internal amino modifier with C6 linker on thymine (for biotinylation);<br>/5Biosg/ or /3Bio/, biotin modification;<br>/BHQ-2/, Black Hole Quencher 2 modification;<br>/5Cy3/ or /3Cy3Sp/, Cy3 dye modification.	

Supplementary Table 2. DNA oligos for TGT synthesis

DNA oligonucleotides were synthesized and purified (high-performance liquid chromatography) by Biosearch Technologies (BHQ2 quencher conjugated samples) or Integrated DNA Technologies.
